# Prediction cardiovascular deterioration in a paediatric intensive care unit (PicEWS): a machine learning modelling study of routinely collected health-care data

**DOI:** 10.1016/j.eclinm.2025.103255

**Published:** 2025-06-18

**Authors:** Dan Fredman Stein, Michael J. Carter, John Booth, Mark J. Peters, Samiran Ray, Neil J. Sebire, Payam Barnaghi, Mario Cortina-Borja

**Affiliations:** a Faculty of Life Sciences and Medicine, King’s College London, UK; b Data Research Innovation and Virtual Environment (DRIVE), Great Ormond Street Hospital for Children, UK; c Paediatric Intensive Care Unit, Oxford University Hospitals NHS Foundation Trust, UK; d NIHR Great Ormond Street Biomedical Research Centre at UCL, UK; e Paediatric Intensive Care Unit, Great Ormond Street Hospital NHS Foundation Trust, UK; f Infection, Immunity and Inflammation Research and Teaching Department, UCL GOS Institute of Child Health, University College London, UK; g Division of Neurology, Department of Brain Sciences, Imperial College London, UK

**Keywords:** Critical & intensive & emergency care, Paediatric intensive care, Digital health, Artificial intelligence

## Abstract

**Background:**

Paediatric intensive care medicine uses fine granular clinical data that describe substantial patient instability to make high-consequence decisions. However, these decisions are also hindered by clinical experts’ ability to interpret longitudinal data along with recent and gradual changes in the vital sign data. Machine learning aided decisions can improve the identification of patient deterioration. Important prior work has predicted outcomes in paediatric intensive care units (PICUs), but has often used non-time series data without age normalisation. Most current work also aims to predict mortality, not potentially treatable clinical inflection points such as cardiovascular deterioration.

**Methods:**

We extracted telemetry data, alongside laboratory and demographic data, from the Electronic Health Record (EHR) of patients admitted to the general PICU at Great Ormond Street Hospital, London (UK), between 1st April 2019 and 31st April 2021. We engineered deterioration monitoring variables into a smaller feature set using a generalisable pipeline. We calculated trend and variability, and used validated age-normalisation for input variables where appropriate. We compared neural network models, gradient-boosted decision trees (XGBoost), and a logistic regression model to predict cardiovascular deterioration within 12 h (defined as a rise in the paediatric Sequential Organ Failure Assessment (pSOFA) cardiovascular sub-score, rising plasma lactate if lactate ≥2 mmol/l, new extra-corporeal membrane oxygenation (ECMO) requirement, or death). We trained the models on a 70-15-15 percent train-test-validation split. We compared model compositions, including without trend, variability, and frequency of input to smaller models. We investigated feature importance using internal feature importance and Shapley Additive Explanation values. We compared the resulting paediatric intensive care early warning score (PicEWS) with the paediatric Sequential Organ Failure Assessment (pSOFA) score as the gold-standard.

**Findings:**

1167 patients were included out of a possible 1195. The best performing predictive model for PicEWS was XGBoost. PicEWS was able to predict cardiovascular deterioration 90% of the time, with fewer than two false alarms for every true alarm. For this model, the area under the precision-recall curve (AUPRC) was 0.552, and area under the receiver operator curve (AUROC) was 0.949. This outperformed pSOFA, which yielded over 10 false alarms per true alarm, with an AUPRC of 0.150 and AUROC of 0.715. The most important features for PicEWS included blood pressure, physiological markers such as bilirubin, and COMFORT score (a sedation and behavioural score used in paediatric intensive care). Feature variability was key to model performance. We demonstrated predictions on an individual patient to show model utility. The study showed that machine learning models can outperform current clinical best practice approaches. We use our model to provide insights into future improvements in clinical practice.

**Interpretation:**

PicEWS outperforms current clinical modelling approaches to predict cardiovascular deterioration. The proposed data processing pipeline and machine learning method offer a clinically applicable decision-support model using age-stratified normal ranges and feature variability over time for the early detection of clinical deterioration in critically ill children.

**Funding:**

The NIHR Great Ormond Street Biomedical Research Centre at UCL and the 10.13039/501100001279Great Ormond Street Hospital Children's Charity peer-reviewed grant award.

**Research in context**

**Evidence before this study**

Whilst there have been many studies predicting clinical deterioration in adult intensive care units, there are only a small number predicting outcomes in Paediatric Intensive Care Units (PICUs). Of these, most focus on late severe outcomes such as cardiovascular collapse or cardiac arrest or rare irreversible outcomes such as death. None use validated age-stratified normal ranges for vital signs. We performed a search on the PubMed database with the terms (((clinical prediction rule [MeSH Terms]) OR (machine learning [MeSH Terms]) OR (ai artificial intelligence [MeSH Terms]) OR (artificial intelligence [MeSH Terms]) OR (artificial intelligence) OR (predictive model) OR (machine learning)) AND (((children [MeSH Terms]) OR (hospital, paediatric [MeSH Terms]) OR (pediatrics [MeSH Terms]) OR (paediatric) OR (paediatric)) AND ((care, intensive [MeSH Terms]) OR (intensive care unit [MeSH Terms]) OR (critical care [MeSH Terms])) OR ((paediatric intensive care units [MeSH Terms]) OR (PICU) OR (paediatric intensive care)) AND (deterioration) from 2016 to 2024, along with Google Search, and checked references from reviews and other relevant articles.

**Added value of this study**

Using a form of machine learning, XGBoost (a type of gradient boosted decision tree), and age-normalised and feature-engineered data, we derived the paediatric intensive care early warning score (PicEWS) model. Unlike previous studies, we compared performance to an existing gold-standard clinical tool (the paediatric Sequential Organ Failure Assessment score, pSOFA). We show that machine learning models can outperform current clinical best practice approaches, and we provide insights into future improvements in clinical practice. We also show how to enhance existing models by incorporating more relevant features, such as temporal variability.

**Implications of all the available evidence**

The predictions from our model could be used as an adjunctive clinical decision support tool or “safety net” in patients with a high probability of cardiovascular deterioration. Identification of early and potentially treatable inflection points on an individual patient’s illness trajectory can improve outcomes.

## Introduction

Accurate and timely prediction of clinical deterioration can enable implementation of interventions to improve patient outcome. This is likely to be most relevant in emergency, peri-operative and intensive care environments where clinical physiology is frequently unstable. Clinical physiological observations were first incorporated into Early Warning Scores (EWS) three decades ago^1^ and subsequently incorporated into the National Early Warning Score (NEWS2), specifically in adults.^2^ NEWS2 is a simple score that takes clinical observations, assigns a score for each, and then summates these to grade severity of physiological abnormalities at the time of observation. Multiple paediatric equivalents exist,^3^ but are more complex in children, in part due to varying normal physiology by age.^4^^,^^5^ More detailed data are typically available in intensive care unit (ICU) environments including biomarkers of organ dysfunction, such as serum bilirubin and coagulation markers. Clinical observations and biomarkers have been incorporated into several organ dysfunction scores, including the paediatric logistic organ dysfunction score (PELOD),^6^ a paediatric version of the sequential organ failure assessment (pSOFA) score,^7^ and the Phoenix Sepsis Score.^8^ These scores are descriptors of organ dysfunction developed to standardise cohorts of patients for epidemiological and clinical studies but have been informally interpreted as predictors of deterioration.

The Acute Physiology and Chronic Health Evaluation (APACHE-II) score in adult intensive care,^9^ and the Paediatric Index of Mortality (PIM-3) score^10^ (among others) are predictive of the incidence of mortality in cohorts of patients requiring intensive care. However, these contain several non-modifiable variables, such as elective admission status, and do not integrate temporal trends or variability in data. They should, therefore, not be used to predict individual patient deterioration. These scoring systems are also unable to “learn” from the accrual of additional data or from changes in the observed associations of model variables with poor outcomes due to changing clinical practice.

More recently, there has been a focus on using machine learning^11^ to predict clinical deterioration over clinically relevant time frames. Much of this work has been done using large data repositories, with a focus on adult ICU data, due to the availability of continuous monitoring in electronic health records (EHR). Several publicly available benchmark adult datasets have encouraged research in this area, including Medical Information Mart for Intensive Care (MIMIC),^12^ eICU,^13^ CCHIC,^14^ and the (non-publicly available) TIPnet dataset from Italian paediatric (P)ICUs.^15^ A Chinese paediatric equivalent, PIC,^16^ is publicly available. However they report substantially higher in-hospital mortality of 9.2% than equivalent UK or US values of 2–4%,^17^^,^^18^ meaning models built on this data would likely not be useful when applied in UK, US, or other similar settings. Modelling approaches to EHR data include classical statistical models,^19^ hierarchical and tree-based models^20^^,^^21^ and artificial neural networks.^22^^,^^23^ However, the clinical applicability of these models to predict deterioration in children is underexplored due to the lack of applicable published paediatric datasets. Previous work in children has also not used validated age-stratification or normalisation for key variables, such as heart rate, that are known to vary significantly according to age.^4^ Patients from ethnic minorities and those with lower socio-economic status are likely to have higher illness severity, are more likely to be admitted to PICU and have worse outcomes.^24^

Using data from a general PICU, we aimed to develop a predictive model for clinical deterioration in children in PICU using machine learning methods. We used feature engineering (converting noisy and duplicate variables into ‘engineered’ cleaned features) and compared several state-of-the-art machine learning methods against the primary outcome.^20^^,^^22^^,^^23^ We hypothesised that the multiparameter data gleaned from EHR data would improve model performance. Given the rarity of mortality in PICUs (<4% of admitted patients in the UK^17^), we used a composite of cardiovascular deterioration, need for extra-corporeal membrane oxygenation (ECMO) and death as our primary outcome for the development of predictive models. We focused on interpretability to allow this approach to inform the development of future predictive scoring systems for use in clinical practice. We used the Transparent Reporting of a multivariable prediction model for Individual Prognosis Or Diagnosis Artificial Intelligence update (TRIPOD + AI) checklist to ensure we followed reporting best practice.

## Methods

### Data extraction

This was a single-centre retrospective observational study of routinely collected EHR data from a large general PICU at Great Ormond Street Hospital (GOSH), London (UK), with accompanying cardiac PICU. The general PICU provides treatment to all children (including externally transferred newborn infants) without a primary cardiac problem. All data from both the general and cardiac PICUs were included for all patients who were admitted at least once to the general PICU. Patients only admitted to the cardiac PICU were excluded. Patients were excluded if their PICU admission was too short for predictions to be made on the data available (3-h to 12-h look-back periods). Non-identifiable data were extracted from the hospital’s EHR, via the GOSH Digital Research Environment. Data for all patients admitted between 1st April 2019 and 31st April 2021 were used. This period covered the first 14 months of the COVID-19 pandemic in the UK, including many children admitted with multisystem inflammatory syndrome in children (MIS-C) associated with SARS-CoV-2 infection. Time-series telemetry data along with demographic, laboratory, medication administration, ward stay, and episode data were extracted. Data from the whole PICU admission were used, but any data from before or after the admission were removed following interpolation. This included all data available to us. This patient cohort has a higher ethnic minority representation than the UK as a whole, reflecting the higher ethnic minority representation in London than the rest of the UK.^17^ The PICU has a risk-adjusted standardised mortality rate matching that which would be expected given the case mix.^17^

### Ethics

This study was performed with UK Health Research Authority (HRA) approval (reference number: 17/LO/008). It used non-identifiable data from the GOSH digital research environment. As this study used only anonymised, routinely collected machine data from NHS records, individual patient consent was not required under the terms of the ethical approval and in accordance with NHS research governance frameworks for anonymised data.

### Processing and feature engineering

All analysis was performed in Python (version 3.8.1) and R (version 4.0.3), using Python packages pandas version 1.2.4, NumPy 1.19.5, Keras 2.4.3 with TensorFlow 2.5.2, XGBoost 1.4.2, scikit-learn 0.24.2, and R packages rriskDistributions 2.1.2 and childsds 0.8.0. The code for the processing pipeline and machine learning model is available on GitHub (github.com/dfs28/PicEWS).

Data were split into time-series (time varying) and non-time-series (time invariant) data types. Infrequently sampled data types, such as laboratory and blood gas results, were treated as non-time-series data. Time-series data included organ support data, including ventilation status and level of vasopressor and inotrope use, and invasive and non-invasive monitoring data such as heart rate, blood pressure, and capillary refill time. Non-time-series data included demographic and laboratory test data.

865 input variables were engineered using clinical insights and clinically applicable variables into 77 features for use in the input data, with examples below (further details in Supplementary Material). A standardised feature processing and engineering pipeline was used for all patients. Feature processing allowed overlapping features and different measurement devices to be consolidated. For some ordinal variables, significant feature processing was performed. For example, for ventilation status, a 4-part ordered variable was generated with different levels of support (Table 1), using 31 input variables. For vasopressor and inotrope use, the vasoactive inotrope score (VIS), a weighted sum, was calculated,^25^^,^^26^ normalised by bodyweight. Where appropriate, for example oxygen flow, values were bodyweight normalised. Age normalisation for heart and respiratory rate was performed using centiles from Fleming and colleagues.^4^ For blood pressure, normalisation to age and sex was done using data from the National High Blood Pressure working group.^5^^,^^27^ Where reference values for the lower limit of normal for diastolic blood pressure were missing, these were calculated from systolic and mean arterial pressures using the relationship $MAP=\frac{1}{3}\left(SBP-DBP\right)+DBP$. As a sensitivity analysis, models with age-normalised scores were compared to models where no normalisation was performed.

**Table 1:** Feature engineering of ventilatory support through the stratification of constituent variables into a clinically relevant ordinal score.

Level of ventilatory support	Example	Value assigned
No ventilatory support	Breathing room air unassisted	0
Supplemental oxygen only	Oxygen delivered by a facemask	1
Positive pressure airway support	High flow nasal oxygen or non-invasive ventilation	2
Invasive mechanical ventilation	Ventilation delivered by endo-tracheal tube or tracheostomy tube	3

Time-series data were coerced to a minute-by-minute time-frame. Most time-series features appear only slightly more frequently than once per hour following feature engineering (e.g., systolic blood pressure appears 1.48 times per hour on average). However as inputs appear in the data asynchronously, preserving a higher frequency maximises the information encoded in this data. Following this, they were linearly interpolated up to 90 min. The maximum time gap between frequently reported observations is 60 min, thus 90 min gives time for observations to be input slightly late. Imputation was performed where values were missing despite interpolation, and different imputation strategies were taken for different data types. For age-dependent variables such as heart rate and blood pressure, stratified median values were imputed according to age. For missing ventilation status, when values such as the fraction of inspired oxygen (FiO_2_) were also missing, patients were assumed not to require any respiratory support. Similar assumptions were made for other features where presence of recorded values was informative. For features where missingness was uninformative but did not require stratification, for example, oxygenated haemoglobin saturation, median imputation was performed. Supplementary Table S1 shows all time-series features used as model inputs. For the non-age normalised models the relevant non-age normalised feature was substituted.

Low-frequency data, including laboratory tests, and demographic data (e.g., weight, height and age) were included for analysis. For weight and height, age and sex (at birth), adjusted *z*-scores were calculated using the childsds package in R,^28^ and by using the UK 1990 growth standard.^29^ Ethnicity was not used to reduce the risk of biased prediction. This was due to the fact that many patients did not have ethnicity information recorded, and variability was not high in the specified ethnicity data. However, we conducted a post-prediction validation based on the known ethnicity data to ensure that the model did not provide unfair predictions for a sub-group. Whilst there are known healthcare inequalities in likelihood of PICU admission and outcomes in PICU, these appear to reflect baseline disease severity and not disease trajectory.^24^ 294 lab test variables were engineered to 53 input features to consolidate multiple data sources. For lab tests, values were carried forward a maximum of 4 days. Median imputation was performed where values were missing despite this. Supplementary Table S2 shows all non-time-series features used as model inputs.

Data were scaled between 0 and 1, and ranked by percentile. Models with 0–1 scaling were compared to those where no scaling was performed. Data were then split into time windows of 3 h, 6 h and 12 h. Predictions were made from the end of the time window. For example, for a patient admitted to PICU at t = 0 h, for the 6 h lookback window, the first predictions could be made at t = 6 h, and for the 12 h model, these would be predicting deterioration between t = 6 h and t = 18 h.

**Fig. 1:**
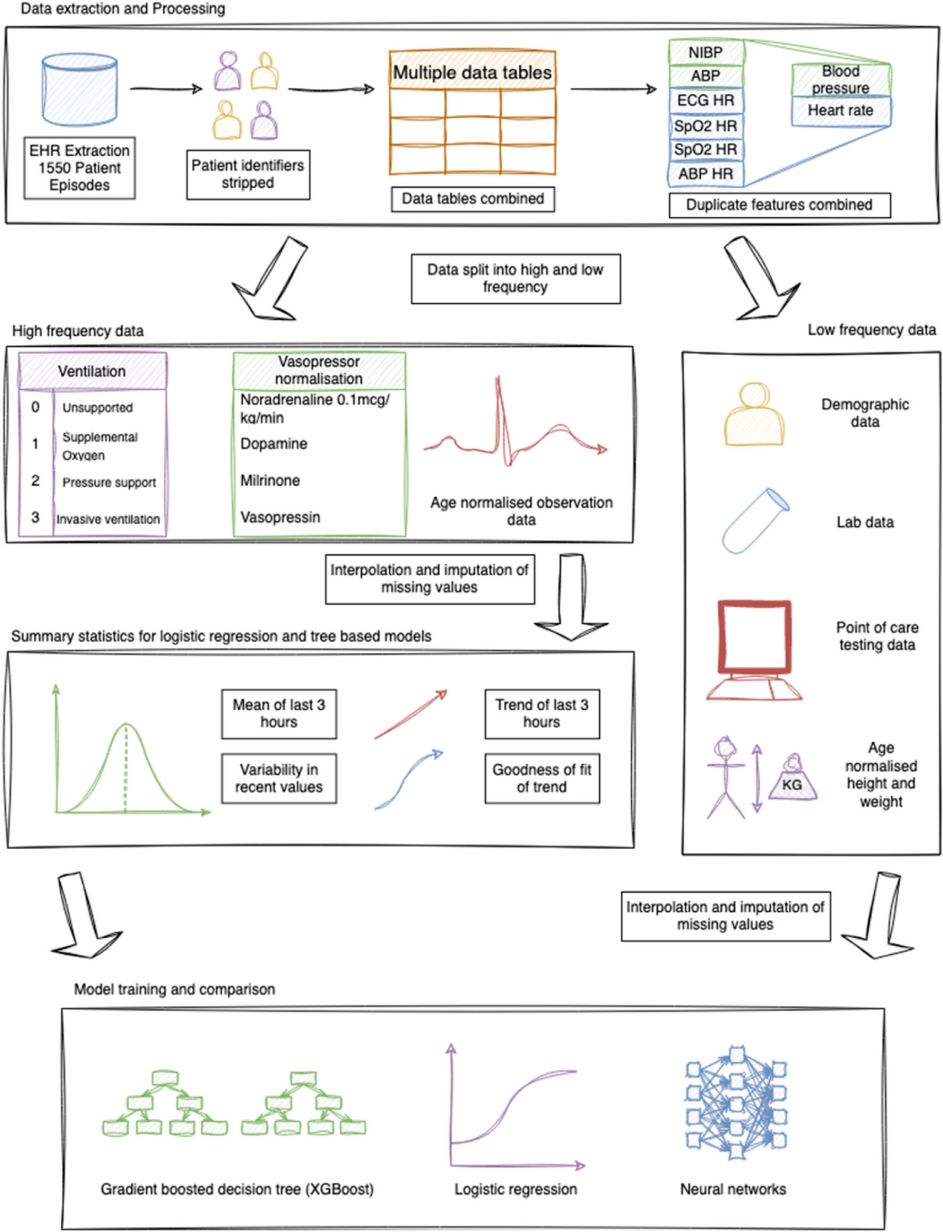
Summary of processing pipeline to make data ready for modelling. Data were extracted and consolidated to reduce input variable number (top panel). Feature engineering was performed, including age normalisation, and summary features produced. Finally, several different models were compared (bottom panel). NIBP: Non-invasive blood pressure, ABP: Arterial blood pressure, EHR: electronic health record, ECG: electrocardiogram, SpO2: oxygenated haemoglobin percentage saturation, HR: heart rate.

To enable the use of the XGBoost and logistic regression models, which take tabular input data, summary features were generated for the time-series data. Mean and standard deviation, trend and strength of trend for each time-series were produced. Trend was calculated by fitting a straight line over the data, and the slope and goodness of fit were used as model inputs. Fig. 1 shows a summary of the overall processing pipeline.

During data processing, data quality was checked using histograms of data distribution, data frequency and feature correlation. These distributions and frequencies were compared to internal audit data from the department and checked with clinicians working in the PICU to ensure that data used were a reliable reflection of the true underlying data.

### Outcomes

The primary outcome was prediction of cardiovascular deterioration. This was defined as a rise in the cardiovascular component of the pSOFA score^30^ (see Supplementary Table S3), plasma lactate ≥2 mmol/l and increasing relative to the previous time-window maximum, new requirement for ECMO, or death. Separate models were built to predict deterioration within 3 h, 6 h, 9 h, 12 h, 18 h and 24 h. The pSOFA cardiovascular component was modified to include milrinone as equivalent to dobutamine. Separate outcomes of death within 48 h and discharge from PICU within 7 days were generated to test multi-outcome neural networks. The primary outcome was chosen by modifying similar criteria used in the literature, for example by Hyland and colleagues^20^ for PICU, as suggested by clinical experts. The cardiovascular component of pSOFA replaced blood pressure and vasopressor and inotrope use in their model; following initiation of vasopressors or inotropes blood pressure is less relevant, and the cardiovascular component of pSOFA gives a stepped score which ensures only clinically relevant changes are captured.

### Models

Three different model types were implemented and compared. These were neural network models, logistic regression and a gradient boosted decision tree (implemented with XGBoost). The neural network models were implemented in Keras with TensorFlow.^31^ Long short-term memory (LSTM) networks, 1- and 2-dimensional convolutional networks, temporal convolutional^32^ and temporal pointwise convolutional networks (TPCN) were built^23^ (further details in Supplementary Materials). The model choice reflected the current state-of-the-art in healthcare time-series models.^21^^,^^23^^,^^32^^,^^33^ Logistic regressions used an L2 weight-penalty, implemented in scikit-learn. The objective function used for XGBoost was area under the precision-recall curve (AUPRC). Model outputs were the probability of deterioration within the specified time-window.

For the neural networks, a 70-15-15 percent train-validation-test split was used, whilst for XGBoost and the logistic regression, an 85-15 percent train-test split was used. Patients were randomised to the different sets with no patient appearing in more than one set. For XGBoost, a 4-fold cross-validation hyperparameter search (with a Bayesian search strategy) was used on the training set, optimized against the AUPRC to ensure maximal performance when the sensitivity threshold was adjusted. This included tuning on the parameter ‘scale positive weight’, which penalises the loss function to improve performance on the minority class, similar to a focal loss function.

### Model testing

All final models were trained on the whole training set (combined training and validation sets: 85%) following hyperparameter search set before being tested on the test set. The same holdout test set was used for all models to ensure comparability. This process was repeated 10 times for every model, and the mean and standard deviations for the different outcome measures were then reported. Precision (positive predictive values), recall (sensitivity), F1 (a composite of precision and recall), AUPRC (area under the precision recall curve) and AUROC (area under the receiver operator curve) were reported for all models. For a recall threshold of 0.9, precision and adjusted F1 were also calculated. This recall set-point was chosen as, at this threshold, only 1 in 10 deterioration events would be missed; a false negative rate of 0.1. pSOFA, as a summary measure, and with individual components separately provided to the model, was used as a comparator measure. pSOFA models were generated using both logistic regression and XGBoost to link to outcome. pSOFA was not implemented during the period of data collection and therefore does not function as a post-treatment variable to bias the results.

To further test model performance, models with limited feature inputs were tested. We ranked the internal feature importances from the XGBoost model to test using smaller numbers of feature inputs. We also tested models where the frequency of input was not included as a feature, models where only mean and variability (not trend) were provided to the model, and models where variables were sampled at a lower frequency (every 15 min and every hour). Hyperparameter search optimisation for these additional experiments was not performed due to computational cost constraints.

To consider interpretability, XGBoost internal feature importance and Shapley Additive Explanation (SHAP) values^34^ were calculated. These describe the contribution of each individual variable to the prediction of the outcome. To visualise these, SHAP importance and waterfall plots were produced.

A study protocol was not prepared prior to publication, and the study was not registered. No patient and public involvement work was performed prior to the study, however clinician end-users were consulted during the design and result producing phases.

### Role of the funding source

The funder had no role in study design, data collection, data analysis, interpretation or writing of this manuscript.

## Results

A total of 1195 patients were eligible for inclusion across 1550 individual admissions. There were 28 patients across 53 admissions in the final 6 h lookback dataset excluded due to having admissions too short for any predictions to be made. There were more male than female patients (657, 56%, vs. 509, 44%) with one patient marked as intersex. The median age of the cohort was 2.29 years, interquartile range 0.43–8.87 years. Demographic data of the patient cohort are detailed in Table 2.

**Table 2:** Patient characteristics, including age, sex, ethnicity, and presence of different outcomes across the test and training sets for the 6 h lookback dataset.

	Training set	Test set
Total patients	1001	166
Male sex	569 (56.8%)	88 (53.0%)
Age	2.28 (IQR 0.43–8.88)	2.56 (0.41–8.79)
Ethnicity		
White	375 (37.5%)	59 (35.5%)
Black	79 (7.9%)	32 (19.3%)
Asian	174 (17.4%)	23 (13.9%)
Other	150 (15.0%)	19 (11.4%)
Prefer not to say	59 (5.9%)	8 (4.8%)
Not recorded	169 (16.9%)	37 (22.2%)
Deteriorated during admission	238 (23.8%)	40 (24.1%)
Required ECMO during admission	2	0
Total 6 h samples	28,942	5137
Median samples per encounter	9.0 (IQR 5.0–28.0)	12.0 (5.0–28.0)
Deteriorated within 12 h	1988 (6.9%)	315 (6.9%)
Rise in lactate within 12 h (>2 mmol/L)	573 (2.0%)	110 (2.1%)
Rise in pSOFA (Cardiac) within 12 h	1411 (4.9%)	203 (4.0%)
Died within 12 h	8	2
New ECMO within 12 h	0	0
Median 6 h average pSOFA (Cardiac)	0 (IQR 0–0.05)	0 (0–0.25)
Serum sodium (mmol/L)	141.8 (95% CI 134–153)	141.2 (134–152.5)
Serum bilirubin (micromol/L)	19.0 (95% CI 2.0–91.0)	18.9 (2.0–74.8)
Serum chloride (mmol/L)	106.5 (95% CI 95.0–119.0)	105.5 (94.3–119.8)
Strong ion gap (MEq/L)	−1.89 (95% CI -2.61 to −1.22)	−1.94 (−2.56 to −1.38)
Mean pSOFA score	5.2 (95% CI 3.0–9.8)	5.4 (3.0–9.9)
Mean comfort score	18.4 (95% CI 13.5–20.0)	18.5 (13.2–20.3)
Mean MAP (mmHg)	65.4 (95% CI 44.8–85.0)	65.6 (44.1–83.0)

IQR: Interquartile range, CI: confidence interval, ECMO: extra-corporeal membrane oxygenation, pSOFA: paediatric Sequential Organ Failure Assessment score, MAP: mean arterial pressure.

All models were compared for prediction of deterioration from the immediate end of the look-back period until 12 h following the look-back period, as this was considered to be the most clinically useful timepoint for prediction. Of the models tested, XGBoost outperformed the neural networks, with the logistic regression second best. At this timepoint, the area under the precision-recall curve (AUPRC) of the XGBoost based model with a 6 h lookback was 0.552 (AUROC 0.949), with the logistic regression AUPRC 0.457 (AUROC 0.923). The best performing neural network used the 3 h lookback, with the best model, the TPCN, having an AUPRC of 0.411 (AUROC 0.923). The neural networks performed better with a shorter look-back period (3 h), and those predicting a single outcome performed better than the multi-outcome networks in most look-back period to output configurations. XGBoost outperformed the neural networks on all other metrics. Supplementary Table S5 shows the final hyperparameters for the XGBoost model with 6 h lookback predicting until 12 h following the look-back period.

**Fig. 2:**
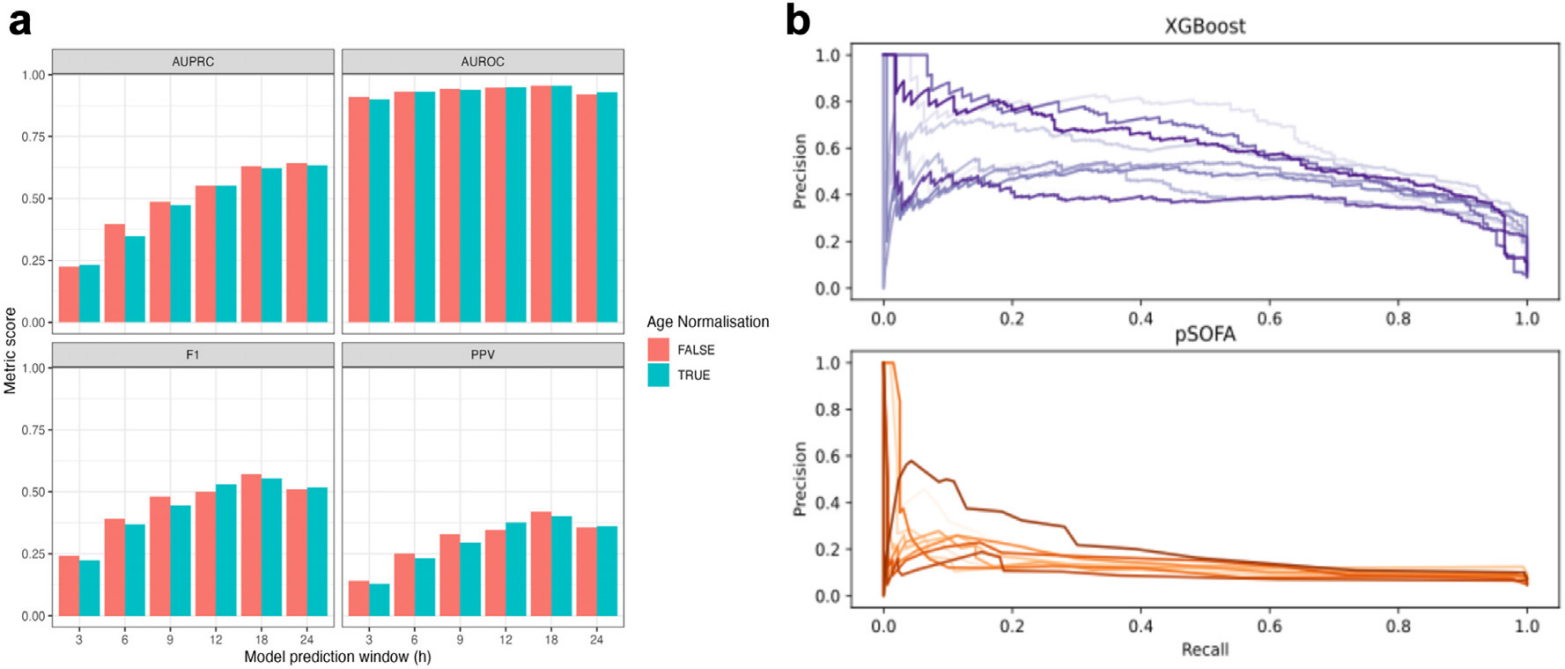
(a) Performance metrics for the XGBoost models using different prediction windows. (b) Comparison of the PRC for the XGBoost model (top) and pSOFA with logistic regression model (bottom). These were produced by performing 10-fold cross-validation on the full dataset (including repeating the hyperparameter search each time) and the out of sample PRCs were then plotted. Across the full range of recall values the precision is substantially greater for the XGBoost model compared to pSOFA (with logistic regression link). AUPRC: Area under the precision-recall curve, AUROC: Area under the receiver-operator curve, F1: a weighted metric including precision and recall, PPV: Positive predictive value, PRC: Precision-recall curve, XGBoost: a type of gradient boosted decision tree, pSOFA: paediatric Sequential Organ Failure Assessment Score.

We compared the performance of pSOFA as a single compound variable against the components of the pSOFA score separately in a logistic regression (Fig. 2). The logistic regression link model for pSOFA performed similarly to the XGBoost link model. The AUPRC of the logistic regression model using pSOFA was 0.150 (AUROC 0.715). This improved to 0.199 (AUROC 0.814) if the individual components were provided separately to the model. This further improved to 0.234 (AUROC 0.842) when trend and variability for the components of pSOFA were provided. Full results for these models are shown in Table 3.

**Table 3:** Results for different models and those with more limited feature inputs**, using a 6 h lookback unless otherwise specified, and predicting deterioration within 12 h.**

	AUPRC	AUROC	Adjusted F1	Adjusted precision
XGBoost with age normalisation	0.552 (0.552–0.552)	0.949 (0.949–0.949)	0.530 (0.530–0.530)	0.375 (0.375–0.375)
XGBoost no age normalisation	0.552 (0.552–0.552)	0.947 (0.947–0.947)	0.500 (0.500–0.500)	0.346 (0.346–0.346)
TPCN (3 h input)	0.411 (0.395–0.427)	0.926 (0.923–0.928)	0.363 (0.305–0.421)	0.270 (0.258–0.283)
Logistic regression	0.457 (0.457–0.457)	0.923 (0.923–0.923)	0.366 (0.366–0.366)	0.230 (0.230–0.230)
pSOFA (LR)	0.150 (0.150–0.150)	0.715 (0.715–0.715)	0.157 (0.157–0.157)	0.085 (0.085–0.085)
pSOFA with individual components (LR)	0.199 (0.199–0.199)	0.814 (0.814–0.814)	0.224 (0.224–0.224)	0.128 (0.128–0.128)
pSOFA with mean + variability (LR)	0.234 (0.234–0.234)	0.842 (0.842–0.842)	0.244 (0.244–0.244)	0.141 (0.141–0.141)
XGBoost latest value only	0.199 (0.199–0.199)	0.827 (0.827–0.827)	0.238 (0.238–0.238)	0.137 (0.137–0.137)
XGBoost without frequency of observation	0.488 (0.488–0.488)	0.940 (0.940–0.940)	0.471 (0.471–0.471)	0.319 (0.319–0.319)
XGBoost without trend	0.518 (0.518–0.518)	0.945 (0.945–0.945)	0.495 (0.495–0.495)	0.341 (0.341–0.341)
XGBoost sampled 15 min	0.442 (0.442–0.442)	0.935 (0.935–0.935)	0.419 (0.419–0.419)	0.273 (0.273–0.273)
XGBoost only sampled hourly	0.397 (0.397–0.397)	0.921 (0.921–0.921)	0.401 (0.401–0.401)	0.258 (0.258–0.258)
XGBoost 20 input features	0.326 (0.326–0.326)	0.890 (0.890–0.890)	0.319 (0.319–0.319)	0.194 (0.194–0.194)
XGBoost 70 input features	0.490 (0.490–0.490)	0.934 (0.934–0.934)	0.492 (0.492–0.492)	0.338 (0.338–0.338)

In brackets we report the 95% confidence intervals. LR: Logistic regression, AUPRC: area under the precision-recall curve, AUROC: area under the receiver operator curve, adjusted F1: a composite of precision and recall, and precision when recall set to 90%.

The best performing XGBoost model incorporated features including variability and trend. Model performance for different predictive windows is shown in Table 4 and Fig. 2. In general, models with a 6 h look-back period performed better than those with a 12 h look-back period (Table 4). For the models with 6 h lookback windows, the results are as follows. The model for predicting deterioration in the following 6 h had an AUPRC of 0.348, an AUROC of 0.932, a precision of 0.232 and F1 of 0.368 at recall 0.9. The model for predicting deterioration in the following 12 h had an AUPRC of 0.552, an AUROC of 0.949, a precision of 0.375 and F1 of 0.530 at recall 0.9. The best performing model was the model for prediction deterioration in 18 h, with an AUPRC of 0.623, an AUROC of 0.956, a precision of 0.401 and F1 of 0.555 at recall 0.9. The models for predicting deterioration in the following 12 h and longer would mean fewer than two false alarms for every true alarm at this high level of recall (sensitivity), missing only 1 in 10 deterioration events.

**Table 4:** Results for XGBoost models with different predictive windows, using different lookback periods, for precision (positive predictive value) and F1 (composite value of precision and recall) when recall (sensitivity) set to 90%.

Predictive Window	3 h	6 h	9 h	12 h	18 h	24 h
**3-h Input**						
With age normalised cutoffs						
AUROC	0.905	0.924	0.937	0.938	0.949	0.909
AUPRC	0.267	0.368	0.446	0.491	0.539	0.516
Positive predictive value	0.111	0.232	0.292	0.338	0.407	0.202
Adjusted F1	0.197	0.342	0.441	0.492	0.560	0.330
Without age normalised cutoffs						
AUROC	0.903	0.923	0.929	0.937	0.945	0.915
AUPRC	0.251	0.364	0.391	0.445	0.503	0.532
Positive Predictive Value	0.155	0.238	0.287	0.346	0.424	0.235
Adjusted F1	0.264	0.377	0.435	0.500	0.567	0.373
**6-h Input**						
With age normalised cutoffs						
AUROC	0.901	0.932	0.939	0.949	0.956	0.929
AUPRC	0.232	0.349	0.473	0.552	0.623	0.634
Positive predictive value	0.128	0.232	0.295	0.375	0.401	0.362
Adjusted F1	0.224	0.368	0.445	0.530	0.555	0.517
Without age normalised cutoffs						
AUROC	0.911	0.932	0.943	0.947	0.956	0.921
AUPRC	0.225	0.396	0.486	0.552	0.630	0.643
Positive predictive value	0.140	0.250	0.328	0.346	0.419	0.356
Adjusted F1	0.242	0.391	0.481	0.500	0.572	0.510
**12-h input**						
With age normalised cutoffs						
AUROC	0.816	0.874	0.887	0.902	0.914	0.911
AUPRC	0.152	0.326	0.387	0.479	0.550	0.614
Positive predictive value	0.094	0.182	0.254	0.329	0.413	0.455
Adjusted F1	0.170	0.304	0.396	0.482	0.567	0.605
Without age normalised cutoffs						
AUROC	0.813	0.881	0.886	0.899	0.914	0.906
AUPRC	0.141	0.334	0.398	0.455	0.591	0.654
Positive predictive value	0.070	0.170	0.267	0.337	0.398	0.454
Adjusted F1	0.129	0.286	0.412	0.491	0.552	0.604

AUPRC: area under the precision-recall curve, AUROC: area under the receiver-operator curve.

Confidence intervals were not reported as XGBoost was highly numerically stable when run multiple times.

We tested different models using XGBoost combined with more limited feature inputs (Table 3). The model without frequency of input performed slightly worse than the overall model (AUPRC 0.488, AUROC 0.940). While frequency of input can be a useful proxy for clinician concern, as patients who have greater monitoring may be already considered more likely to deteriorate, the models still perform well without it. The model where trend and strength of trend were not provided also performed well (AUPRC 0.517, AUROC 0.945), suggesting trend has some limited utility. However, the model where latest value was used alone, performed poorly without variability (AUPRC 0.199, AUROC 0.827).

Models without age normalisation were also tested. These performed similarly to the models with age-normalised inputs. Specifically, the non-age normalised model a 6 h look-back and prediction of deterioration in the following 12 h had an AUPRC 0.552 (AUROC 0.947) (Fig. 2a, Table 4).

We investigated whether frequency of inputs would affect variability and therefore patients with more intensive monitoring because of clinician concern would have artificially higher variability. We sampled the time-series data at lower frequencies (15 min and once per hour) and calculated mean and standard deviation across these time-periods (Table 3). The model still performed well with lower frequency input data, with the 15-min model AUPRC 0.442 (AUROC 0.935) and hourly model AUPRC 0.397 (AUROC 0.921).

Models with fewer input features were also tested (Table 3). The model with the top 70 features performed close to the full model (AUPRC 0.490, AUROC 0.934). The model with only 20 features had degraded performance, however (AUPRC 0.326, AUROC 0.890), with more false positives at the high set point of sensitivity (precision 0.268 vs. 0.342 for the 70-feature model).

**Fig. 3:**
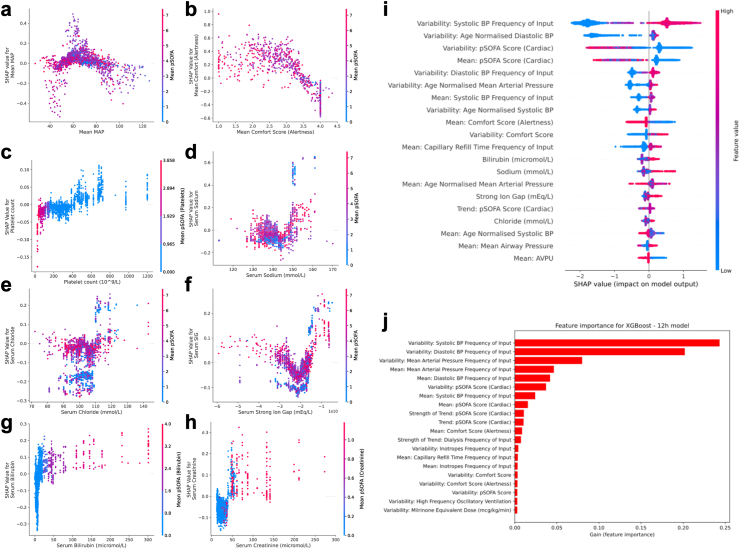
SHAP values for mean arterial pressure (MAP), mean COMFORT score (sedation level), platelet count, serum sodium, serum chloride, serum strong ion gap (SIG, a measure of metabolic acidosis), serum bilirubin and serum creatinine (a–h). SHAP values plotted against input feature values for the top 20 values (i) and XGBoost internal feature importance for the top 20 features (j). SHAP: SHapley Additive exPlanation values, COMFORT: a behaviour and sedation score used in paediatric intensive care, XGBoost: a type of gradient boosted decision tree.

We used the internal feature importance and SHAP values to interrogate the XGBoost model. We compared models with and without age-normalised values to investigate the differences in model predictions (Fig. 3). The most important features relate to blood pressure (BP, including the cardiovascular component of pSOFA), including variability and frequency of input, for models with and without age-normalisation. For the internal feature importances, both models use similar inputs to make predictions, including those related to BP, VIS and individual BP support metrics, capillary refill time, COMFORT score (a sedation and behavioural score, Supplementary Table S4) and pSOFA. Both age-normalised and non-age-normalised models use a mix of summary measures over the 6 h input window, including mean, variability, and frequency of input. The model with age-normalised inputs also identified laboratory values previously associated with illness severity, including strong ion gap, serum bilirubin, and creatinine, the latter two of which are included within the total pSOFA (Fig. 3). Of note, frequency of input in several features, including capillary refill time and BP was inversely associated with the probability of prediction of deterioration. SHAP values plotted against the input values (Fig. 3a–h) demonstrated the ability of XGBoost to account for non-linear relationships (Fig. 3c, g, h), and complex interactions between features (3 d-f). Fig. 3c shows how individual features, in this case platelet count, were used differently by the XGBoost models in comparison with their use in pSOFA, where a low platelet count results in a high pSOFA score.

**Fig. 4:**
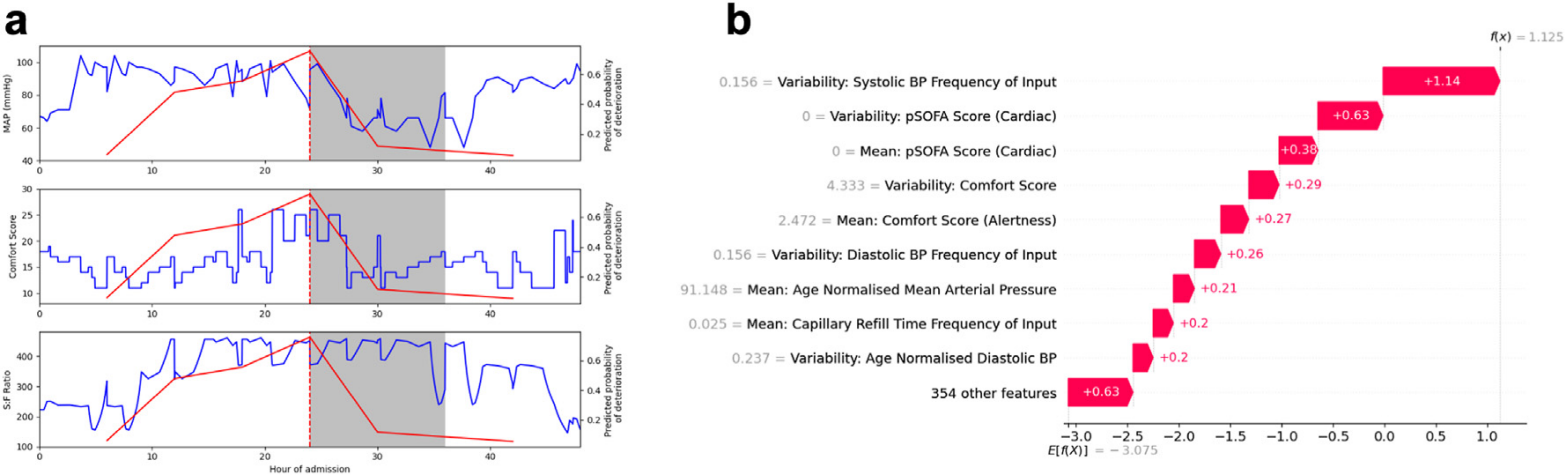
Predicted probability of deterioration. (a) PicEWS model output plotted alongside mean arterial pressure (MAP), COMFORT Score and S:F Ratio, plotted against hour of admission. The red vertical line indicates the highest probability of deterioration and the grey box indicates the prediction interval. (b) SHAP values alongside the raw input values for this prediction. PicEWS: Paediatric Intensive Care Early Warning Score, MAP: Mean arterial Pressure, COMFORT score: a behaviour and sedation score used in paediatric intensive care, S:F Ratio: Oxygenated Haemoglobin Saturation (SpO2) to fraction inhaled oxygen (FiO2) ratio, SHAP: SHapley Additive exPlanation values.

We illustrated the model in individual patients to demonstrate the value in clinical practice of PicEWS being used. We plotted the raw predictions against their clinical data over time (Fig. 4a) and took the SHAP values for this prediction (Fig. 4b). In this individual patient, the probability of deterioration in three features increased from <10% to >75% over a 24 h period immediately prior to a cardiovascular deterioration (mean arterial pressure, MAP; COMFORT score; and the fraction of inhaled oxygen required to maintain a level of oxygen saturation, S:F ratio).

## Discussion

We developed and validated a paediatric intensive care early warning score (PicEWS) model to predict cardiovascular deterioration accurately in 1167 patients over 1497 individual admissions, agnostic to patient diagnosis, using real-world data. The cohort consisted of children with a median age of 2.29 with diverse critical illnesses in a large general PICU in the UK. We used cardiovascular deterioration as the primary outcome. We assessed neural networks, XGBoost and logistic regression models using features engineered from several hundred variables, and compared these models to those derived from total pSOFA (and its component variables) alone.

Several disease severity scores have been implemented to describe organ dysfunction, such as pSOFA^30^ and the Phoenix Sepsis Score.^35^ Although these scores are calibrated by an association with increased mortality,^8^ they are not designed as predictive scores, although they may informally be (mis)interpreted as such in clinical practice. Predictive scoring systems, such as PIM-3 are suitable for predicting the incidence of mortality in cohorts of children requiring PICU admission but are not optimised for individual patient predictions and contain several non-modifiable, non-physiological, variables such as elective admission status. (The only demographic variables implemented in PicEWS were age, weight and sex at birth.) These scoring systems use a single input variable per category, with no account of variability or trend. In contrast, the PicEWS model described here used a 6 h look-back and 12 h prediction periods to accurately predict deterioration in children in intensive care, taking account of trends in the data. We set the recall (sensitivity) of the model to 0.9 (90%) as a pragmatic approach to balance the clinical need avoid “missing” deteriorating patients against the risk of “alarm fatigue” in a non-specific score. Our use of cardiovascular deterioration, rather than death or length of stay,^21^^,^^23^ allowed us to focus on a clinically useful outcome that could be potentially modifiable with interventions. To aid clinical utility, the model reports the prediction of deterioration (with associated data on precision) and has an interpretability feature that describes the feature-specific contribution to the prediction (the SHAP values). We found that model performance remained good using a smaller feature set, without using trend or frequency of inputs, but deteriorated significantly when variability was not included. This shows the need for future scoring systems to integrate both a greater number of input features and variability within a time window, made possible by the increasing ability of EHR systems to automate these scoring systems. Data relating to frequency of reported observation is important to model performance; models where this was not included had a degree of performance degradation. The importance of these types of meta-data has been previously noted, as their presence allows models to ‘look over the shoulders’ of the clinicians looking after patients. This allows the models to predict outcomes from the actions of doctors and other healthcare professionals,^36^ and in some cases the meta-data can be better than the data themselves for predicting outcomes.^37^ For example, where invasive blood pressure monitoring is present, although inputs from this automatically pull through to our dataset, frequency of blood pressure reporting may be higher than non-invasive blood pressure monitoring, showing clinician concern about a patient. This again emphasises the importance of meta-data in model performance. Although the timing of data import is automated, local policies, for example around inotrope and vasopressor use, will determine whether invasive blood pressure monitoring will be present, highlighting again how the data reflects clinician practice and concern.

There has been a large amount of work using machine learning models to predict deterioration in adults,^38^ but limited work in children. Using a literature search we were only able to find a handful of previous studies investigating deterioration in general PICU patients (although there was some further work in cardiac PICU patients^39^). Of the relevant studies, Aczon and colleagues,^40^ Kim and colleagues,^41^ Lee and colleagues^21^ and Potes and colleagues,^42^ had larger datasets (9070, 1445, 2496, and 7052 patients respectively), using a variety of methodologies. However, only Potes and colleagues used deterioration (need for haemodynamic intervention), and had worse results (AUROC 0.81). The other studies used mortality as the outcome for prediction. However, 2–4% of patients admitted to PICU in high income settings die during their admission.^17^^,^^18^^,^^43^ The use of mortality as an outcome may miss patients who have imminent reversible deterioration. Comoretto and colleagues^15^ used an XGBoost model to predict haemodynamic failure, in almost 30,000 patients. However, haemodynamic failure was a rare outcome (∼1% in the dataset) and may not predict earlier and possibly more reversible deterioration. A few smaller studies predicted reversible deterioration: Matam and colleagues^44^ predicted cardiac arrest and Izquierdo and colleagues^45^ predicted deterioration. However, both had worse performance than our model, and Izquierdo and colleagues do not define deterioration. We found that model performance remained good on the validation cohort when smaller training sets were used (Supplementary Figures), suggesting that in a single centre context with good quality data, dataset size may be less relevant.

With PicEWS, we used as many variables as were consistently available. XGBoost gave more precise predictions of outcome in comparison with neural networks, despite being unable to take time series data as input. It also has low computational requirements relative to the neural network models. We showed that higher frequency data input improved model performance. Although the data were available on average only slightly more frequently than once per hour, maintaining the exact temporal ordering seemed to provide more information and therefore better performance than summary statistics provided on less frequently sampled data. Unlike other attempts at using machine learning models of paediatric intensive care data, we used age-normalised values, although this had only limited impact on model accuracy, suggesting that XGBoost in the PicEWS model was able to appropriately adjust features for age. This was supported by other testing data (not shown). Although we used static train-validation-test sets, the ability of the model to “learn” means that PicEWS could be iteratively improved when implemented in the same context, and could also adapt to new contexts, through a retraining and re-validation process. A “learning” PicEWS model would also iteratively automate the need for regular manual review as the association between features and outcome changes (for example with improved therapies). We propose that the data would be presented as part of an interactive dashboard containing relevant input features and patient data, and that predictions are presented as a percentage, requiring minimal training of end users.

This study contains several acknowledged limitations. The model needs to be tested on an external validation set, with a larger number of patients. Following this, validation of the model in different settings – or more accurately, implementation of the model and assessment of any change in the relationship between features and outcome – is required. External validation is especially important to ensure generalisability where population sizes are small, as in the PICU. The model was tested in patients admitted to a general PICU, and implementation in a cardiac surgical PICU and other specialist centres may reveal additional physiological measurements or inputs that are useful for the prediction of deterioration. In addition, the frequency of inputs was one of the strongest predictors of cardiovascular deterioration, requiring more investigation of the cause of the variable recording frequency of these data, although previous research has suggested this represents a reflection of clinical concern about patients.^36^^,^^37^ This was a relatively small dataset, limiting our ability to perform subgroup analysis. It has been previously noted that paediatric patients can have a wide range of clinical phenotypes even when the physiological trigger is the same,^46^ and understanding the heterogeneity of organ dysfunction across different subgroups will be required for future trials. We aimed to address this in Fig. 3, but future external validation sets will be required to allow further investigation. Finally, we used cardiovascular deterioration as primary outcome: 23.8 percent of children had this outcome during their PICU admission. We chose cardiovascular deterioration since it is on a pathway towards physiological deterioration and, ultimately, death in critically ill children, but it may be reversible given sufficient prediction. However, in some cases, this deterioration may not be reversible, or the treatment itself, for example, vasoactive medications, is contained within the outcome score, such as pSOFA. We propose to extend the primary outcome to other organ system dysfunctions, such as respiratory failure, in future iterations of PicEWS. Finally, all machine learning models rely on the quality and quantity of their input data. Our approach is, therefore, only suitable for the minority of high-resource settings that have implemented high-frequency EHR data collection in critically ill children.^47^ It should also be used with caution where only a small number of the input features are available.

In conclusion, we were able to predict cardiovascular deterioration in critically ill children in PICU using a gradient-boosted decision tree-based (XGBoost) machine learning model (PicEWS). The model was precise: when set at a recall (sensitivity) of 0.9 led to fewer than two false alarms per real deterioration. It outperformed existing scores, and we were able to show that a larger number of relevant inputs, higher frequency sampling, feature variability and frequency of input all improve model performance. To aid interpretability and, therefore, intervention to prevent imminent deterioration, PicEWS presents a rolling prediction of deterioration over time, with precision estimates, and details the features contributing most to the prediction of deterioration. This may be suitable for integration within EHR interfaces within a single-centre, and ultimately multicentre, trial of the model as a decision-support tool for clinicians caring for critically ill children.

## Contributors

DFS, MCB, MJC and PB conceptualised the project. DFS, NS and JB curated the data. DFS and MJC analysed the data. DFS, MCB, PB, MJC, SR and MJP worked on the methodology. MCB, PB and MJC provided supervision. DFS and MJC wrote the manuscript. MCB, MJP, PB, SR and NS reviewed the manuscript. All authors read and approved the final version of the manuscript. DFS and JB have directly accessed and verified the underlying data.

## Data sharing statement

Due to privacy and information governance the data for this study cannot be made publicly available due to information governance constraints. Source code for the analysis is available at github.com/dfs28/PicEWS. Model hyperparameters are included in the Supplementary materials.

## Declaration of interests

DFS would like to acknowledge a UK National Institute for Health and Care Research (NIHR) Academic Clinical Fellowship, and a grant from the Centre for Ageing and Resilience in a Changing Environment at King’s College London.

SR would like to declare NIHR Health Technology Assessment (HTA) funding as part of the Oxy-PICU and PRESSURE trials, UKRI Engineering and Physical Sciences Research Council (EPSRC) funding as part of the University College London CHIMERA hub, EU Horizon and UK Research and Innovation (UKRI) funding as part of the Phems project, and from La Roche Ltd for consulting fees for educational materials.

PB would like to acknowledge funding via the Royal Academy of Engineering and Great Ormond Street Hospital, the UK Dementia Research Institute (award number UK DRI-7002) through UK DRI Ltd, principally funded by the Medical Research Council, and the UKRI Engineering and Physical Sciences Research Council (EPSRC) and the National Institute of Health and Care Research (NIHR) (grant number: EP/W031892/1).

MJP would like to declare grant funding from NIHR HTA for clinical trials in critically ill children, payment for expert testimony in criminal and medical negligence cases. He is also Deputy Chair of the NIHR HTA General Funding committee.

MJC would like to declare funding from the NIHR for an Academic Clinical Lectureship.
