# Supplementary material for: Prediction cardiovascular deterioration in a paediatric intensive care unit (PicEWS): a machine learning modelling study of routinely collected health-care data
Source: eClinicalMedicine. 2025 Jun 18;85:103255. doi: 10.1016/j.eclinm.2025.103255 (PMC12213932; doi:10.1016/j.eclinm.2025.103255)
Supplement: Supplementary Figs. S1 and S2 and Tables S1–S5 [file mmc1.docx]

**Supplementary Materials:**

Supplementary Figure 1: Performance of the XGBoost model according to the number of input features. AUPRC: area under the precision recall curve, AUROC: area under the receiver operator curve.

Ai Aii

Bi Bii

Ci Cii

Ei Eii

Di Dii

Supplementary Figure 2: Neural network structures. Ai: Long short-term memory (LSTM) network with multiple simultaneous output prediction. Aii: LSTM with single output prediction. Bi: 2-dimensional convolutional neural network (2D CNN) with multiple simultaneous output prediction. Bii: 2D CNN with single output prediction.

Ci: 1-dimensional CNN with multiple simultaneous output prediction. Cii: 1-dimensional CNN with single output prediction.

Di: Temporal Convolutional Network (TCN) with multiple simultaneous output prediction. Dii: TCN with single output prediction. Ei: Temporal Pointwise Convolutional Network (TPCN) with multiple simultaneous output prediction. Eii: TPCN with single output prediction.

Supplementary Figure 3: Performance of the XGBoost model according to the number of training samples. AUPRC: area under the precision recall curve, AUROC: area under the receiver operator curve.

| Non-time-series input features |  |
| --- | --- |
| ALT (IU/L) | Monocytes (x10^9/L) |
| Albumin (g/L) | Neutrophils (x10^9/L) |
| Alkaline Phosphatase (U/L) | P50 (mmHg) |
| AST (IU/L) | PaCO2 (kPa) |
| Aspartate (U/L) | PcCO2 (kPa) |
| Amylase (U/L) | PmCO2 (kPa) |
| APTT (s) | PaO2 (kPa) |
| Anion gap | PcO2 (kPa) |
| Base excess (mEq/L) | PmO2 (kPa) |
| Basophils (x10^9/L) | PO2 (kPa) |
| Bicarbonate (mmol/L) | PvCO2 (kPa) |
| pH | Phosphate (mmol/L) |
| Blood culture performed | Platelets (x10^9/L) |
| Creatinine (micromol/L) | Potassium (mmol/L) |
| CRP (mg/L) | PT (s) |
| Calcium (mmol/L) | Reticulocytes (%) |
| Chloride (mmol/L) | Sodium (mmol/L) |
| Eosinophils (x10^9/L) | TT (s) |
| Fraction of Deoxyhaemoglobin | Bilirubin (micromol/L) |
| Methemoglobin (%) | White Cell Count (x10^9/L) |
| Fraction of Oxygenated Haemoglobin | Strong Ion Gap (mEq/L) |
| Glucose (mmol/L) | Age (years) |
| HCT (%) | Sex |
| Haematocrit (L/L) | Weight (kg) |
| INR | Height (m) |
| Lactate (mmol/L) | Age Normalised Weight |
| Lymphocytes (x10^9/L) | Age Normalised Height |
| Magnesium (mmol/L) |  |

Supplementary table 1: Non-time-series model input features

| Time series input features |  |
| --- | --- |
| Ventilation Status | Capillary Refill Time Frequency of Input |
| High Frequency Oscillatory Ventilation | Oxygen Saturation (%) |
| Tracheostomy Status | Oxygen Saturation Frequency of Input |
| End Tidal CO2 (mmHg) | Height (m) |
| FiO2 | Extra-Corporeal Membrane Oxygenation Status |
| Oxygen Flow Rate (L/min) | Extra-Corporeal Membrane Oxygenation Status Frequency of Input |
| Weight Adjusted Oxygen Flow Rate (L/min/kg) | Vasoactive Inotropic Score (mcg/kg/min) |
| IPAP (cmH2O) | Vasopressin Equivalent dose (mcg/kg/min) |
| Ventilation (ml/min) | Dopamine Equivalent Dose (mcg/kg/min) |
| Mean Airway Pressure | Total Noradrenaline and Adrenaline Dose (mcg/kg/min) |
| EPAP (cmH2O) | Milrinone Equivalent Dose (mcg/kg/min) |
| Ventilation Status Frequency of Input | Inotropes Frequency of Input |
| Oxygen Flow Rate Frequency of Input | Vasopressin Frequency of Input |
| IPAP Frequency of Input | Dopamine Frequency of Input |
| EPAP Frequency of Input | Noradrenaline and Adrenaline Frequency of Input |
| FiO2 Frequency of Input | Milrinone Frequency of Input |
| High Frequency Oscillatory Ventilation Frequency of Input | SpO2:FiO2 ratio |
| Tracheostomy status Frequency of Input | pSOFA Score (Respiratory) |
| Ventilation (ml/min) Frequency of Input | pSOFA Score (Cardiac) |
| Mean Airway Pressure Frequency of Input | pSOFA Score (Platelets) |
| End Tidal CO2 Frequency of Input | pSOFA Score (Bilirubin) |
| Systolic BP Frequency of Input | pSOFA Score (GCS) |
| Diastolic BP Frequency of Input | pSOFA Score (Creatinine) |
| Mean Arterial Pressure Frequency of Input | Respiratory Rate Frequency of Input |
| Heart Rate Frequency of Input | Dialysis Status |
| Comfort Score (Alertness) | Dialysis Frequency of Input |
| Comfort Score (BP) | pSOFA Score (Total) |
| Comfort Score (Calmness) | Temperature (F) |
| Comfort Score (Total) | Temperature Frequency of Input |
| Comfort Score (HR) | Urine Output (ml) |
| Comfort Score (Resp) | Urine Output (ml/kg) |
| AVPU | Urine Output Frequency of Input |
| GCS (Verbal) | Paediatric Early Warning Score (Total) |
| GCS (Eyes) | Age Normalised Heart Rate |
| GCS (Motor) | Age Normalised Respiratory Rate |
| GCS (Total) | Age Normalised Diastolic BP |
| GCS Frequency of Input | Age Normalised Systolic BP |
| AVPU Frequency of Input | Age Normalised Mean Arterial Pressure |
| Capillary Refill Time (s) | |

Supplementary table 2: Time series-input features

| **Variables** | **Score** |  |  |  |  |
| --- | --- | --- | --- | --- | --- |
| **0** | 1 | 2 | 3 | 4 |  |
| **Respiratory** |  |  |  |  |  |
| **PaO2:FiO2^a^** | ≥400 | 300-399 | 200-299 | 100-199 With respiratory support | <100 With respiratory support |
| **or** |  |  |  |  |  |
| **SpO2:FiO2** | ≥292 | 264-291 | 221-264 | 148-220 With respiratory support | <148 With respiratory support |
| **Coagulation** |  |  |  |  |  |
| **Platelet count, ×10^3^ /μL** | ≥150 | 100-149 | 50-99 | 20-49 | <20 |
| **Hepatic** |  |  |  |  |  |
| **Bilirubin, mg/dL** | <1.2 | 1.2-1.9 | 2.0-5.9 | 6.0-11.9 | >12.0 |
| **Cardiovascular** |  |  |  |  |  |
| **MAP by age group or vasoactive infusion, mm Hg or μg/kg/min^b^** | | | |  |  |
| **<1 mo** | ≥46 | <46 | Dopamine hydrochloride ≤5 or dobutamine hydrochloride (any) | Dopamine hydrochloride >5 or epinephrine ≤0.1 or norepinephrine bitartrate ≤0.1 | Dopamine hydrochloride >15 or epinephrine >0.1 or norepinephrine bitartrate >0.1 |
| **1-11 mo** | ≥55 | <55 |  |  |  |
| **12-23 mo** | ≥60 | <60 |  |  |  |
| **24-59 mo** | ≥62 | <62 |  |  |  |
| **60-143 mo** | ≥65 | <65 |  |  |  |
| **144-216 mo** | ≥67 | <67 |  |  |  |
| **>216 mo** | ≥70 | <70 |  |  |  |
| **Neurologic** |  |  |  |  |  |
| **Glasgow Coma Score^c^** | 15 | 13-14 | 10-12 | 06-09 | <6 |
| **Renal** |  |  |  |  |  |
| **Creatinine by age group, mg/dL** | |  |  |  |  |
| **<1 mo** | <0.8 | 0.8-0.9 | 1.0-1.1 | 1.2-1.5 | ≥1.6 |
| **1-11 mo** | <0.3 | 0.3-0.4 | 0.5-0.7 | 0.8-1.1 | ≥1.2 |
| **12-23 mo** | <0.4 | 0.4-0.5 | 0.6-1.0 | 1.1-1.4 | ≥1.5 |
| **24-59 mo** | <0.6 | 0.6-0.8 | 0.9-1.5 | 1.6-2.2 | ≥2.3 |
| **60-143 mo** | <0.7 | 0.7-1.0 | 1.1-1.7 | 1.8-2.5 | ≥2.6 |
| **144-216 mo** | <1.0 | 1.0-1.6 | 1.7-2.8 | 2.9-4.1 | ≥4.2 |
| **>216 mo^e^** | <1.2 | 1.2-1.9 | 2.0-3.4 | 3.5-4.9 | ≥5 |

Supplementary Table 3: pSOFA Score^1^. Abbreviations: FiO2, fraction of inspired oxygen; MAP, mean arterial pressure; pSOFA, paediatric Sequential Organ Failure Assessment; SpO2, peripheral oxygen saturation. SI conversion factors: To convert bilirubin to micromoles per litre, multiply by 17.104; creatinine to micromoles per litre, multiply by 88.4; and platelet count to ×109/L, multiply by 1.
^a^ PaO2 was measured in millimeters of mercury.
^b^ MAP (measured in millimeters of mercury) was used for scores 0 and 1; vasoactive infusion (measured in micrograms per kiligram per minute), for scores 2 to 4.
^c^ Glasgow Coma Scale was calculated using the paediatric scale.

| **Score** | **1** | **2** | **3** | **4** | **5** |
| --- | --- | --- | --- | --- | --- |
| **Scale** |  |  |  |  |  |
| **Alertness** | Deeply asleep | Lightly asleep | Drowsy | Alert and Awake | Hyper-alert |
| **Calmness/agitation** | Calm | Slightly Anxious | Anxious | Very Anxious | Panicky |
| **Respiratory response** | No coughing or spontaneous respiration | Spontaneous respiration | Occasional cough/resists ventilator | Actively breathes against ventilator | Fights ventilator – coughs/chokes/gags |
| **Physical movement** | None | Occasional, slight movements | Frequent, slight movement | Vigorous movements of extremities only | Vigorous movements of extremities, torso and head |
| **Blood pressure** | BP 15% below baseline | BP consistently at baseline | Infrequent elevations of ≥ 15% (one to three during observation period) | Infrequent elevations of ≥ 15% (more than three during observation period) | Sustained elevation of ≥ 15%. |
| **Heart rate** | Heart rate 15% below baseline | Heart rate consistently at baseline | Infrequent elevations of ≥ 15% (one to three during observation period) | Infrequent elevations of ≥ 15% (more than three during observation period) | Sustained elevation of ≥ 15%. |
| **Muscle tone** | Relaxed/none | Reduced muscle tone | Normal muscle tone | Increased tone/flexion – fingers/toes | Extreme rigidity/flexion – fingers/toes |
| **Facial tension** | Relaxed | Normal tone | Some tension | Full facial tension | Hyper-alert |

Supplementary Table 4: COMFORT Score^2^

References:

1. Matics TJ, Sanchez-Pinto LN. Adaptation and Validation of a Pediatric Sequential Organ Failure Assessment Score and Evaluation of the Sepsis-3 Definitions in Critically Ill Children. JAMA Pediatr. 2017 Oct 2;171(10):e172352.

2. Wolf A, McKay A, Spowart C, Granville H, Boland A, Petrou S, et al. COMFORT score. In: Prospective multicentre randomised, double-blind, equivalence study comparing clonidine and midazolam as intravenous sedative agents in critically ill children: the SLEEPS (Safety profiLe, Efficacy and Equivalence in Paediatric intensive care Sedation) study [Internet]. NIHR Journals Library; 2014 [cited 2024 Dec 2]. Available from: https://www.ncbi.nlm.nih.gov/books/NBK269133/

| Hyperparameter | Value |
| --- | --- |
| colsample_bylevel | 0.96 |
| colsample_bytree | 0.66 |
| gamma | 0.50 |
| learning_rate | 0.01 |
| max_delta_step | 2.00 |
| max_depth | 10.00 |
| min_child_weight | 14.00 |
| n_estimators | 173.00 |
| reg_alpha | 0.00 |
| reg_lambda | 0.00 |
| scale_pos_weight | 0.60 |
| subsample | 0.51 |

Supplementary Table 5: Final hyperparameters for XGBoost following tuning using Bayesian Search Strategy.
